# Microstructure of Croatian Wild Grapevine (*Vitis vinifera* subsp. *sylvestris* Gmel Hegi) Pollen Grains Revealed by Scanning Electron Microscopy

**DOI:** 10.3390/plants11111479

**Published:** 2022-05-31

**Authors:** Katarina Lukšić, Goran Zdunić, Ana Mucalo, Luka Marinov, Zorica Ranković-Vasić, Jelena Ivanović, Dragan Nikolić

**Affiliations:** 1 Institute for Adriatic Crops and Karst Reclamation, Put Duilova 11, 21 000 Split, Croatia; katarina.luksic@krs.hr (K.L.); ana.mucalo@krs.hr (A.M.); luka.marinov@krs.hr (L.M.); 2 Faculty of Agriculture, University of Belgrade, Nemanjina 6, 11080 Belgrade, Serbia; zoricarv@agrif.bg.ac.rs (Z.R.-V.); angelina.ivanovic.95@gmail.com (J.I.); nikolicd@agrif.bg.ac.rs (D.N.)

**Keywords:** *Vitis vinifera* subsp. *sylvestris*, flower morphology, pollen structure, SEM

## Abstract

Wild grapevine (*Vitis vinifera* subsp. *sylvestris* Gmel Hegi) is dioecious with male and female plants, whereas domesticated grapevine is mostly hermaphrodite with self-fertile hermaphrodite flowers. The pollen morphology of wild grapevine has been poorly studied. There is no detailed palynological study of *V. sylvestris* in Croatia and neighboring countries. Here, scanning electron microscopy (SEM) was used to analyze the pollen of *V. sylvestris* from male and female individuals growing at two natural sites in Croatia. The selective APT3 marker was used to confirm the flower phenotype with the genetic background. SEM analysis showed that the pollen grains of *V. sylvestris* were isopolar and radially symmetrical, with foveolate perforated ornamentation, regardless of the flower type of the individuals. All male flowers were 3-colporate and prolate in shape, whereas female individuals varied from subprolate to spheroidal and had inaperturate pollen grains. Pollen shape, dimensions and exine ornamentation proved very informative, and here we address the most polymorphic traits in the analyzed *V. sylvestris* individuals. Principal component analysis (PCA) and clustering based on pollen morphology variables clearly differentiated individuals by their flower type, and no grouping specific to population was observed, pointing to the conserved pollen structure of *V. sylvestris*. The results indicate the need to continue the palynological study of *V. sylvestris* and serve as a good phenotypic basis for functional genetic studies on genes involved in pollen morphology and function.

## 1. Introduction

The Eurasian grapevine (*Vitis vinifera* L.) includes two subspecies: wild (*Vitis vinifera* L. subsp. *sylvestris* Hegi Gmelin, hereafter *V. sylvestris*) and domesticated grapevine (*Vitis vinifera* L. subsp. *vinifera*, hereafter *V. vinifera*). Both subspecies are diploid (2n = 38), sexually compatible, and prefer cross-pollination [1]. Despite sexual compatibility, in practice, spatial dislocation between natural *V. sylvestris* habitats and vineyards and differences in flowering time between the two subspecies often result in low pollen exchange [2].

The wild grapevine is dioecious and presumed to be an ancestor of domesticated grapevines [3]. The crucial difference between the two subspecies is in flower morphology. Domesticated grapevine has mostly hermaphroditic and, in some cultivars, female-type flowers, whereas wild grapevine exhibits male or female flowers. Recent transcriptomic studies showed that all three types of flowers begin their development as perfect hermaphroditic flowers [4,5,6]. In later stages of flower specification, male flowers show a reduced pistil, style and stigma, whereas females have shorter, backward-reflexed or shriveled stamens with sterile pollen [6,7].

Pollen morphology proved important in recent studies, such as archaeobotany [8], taxonomic and morphological characterization [8,9,10] and pollination studies [11]. Due to the great economic importance of grapevine, pollen studies have mainly focused on cultivars and their productivity [12,13,14,15,16,17]. Domesticated and wild grapevine differ significantly in yield [18,19] depending on pollen fertility [1,20,21]. The two subspecies are characterized by pollen dimorphism. Dimorphism showed to be related to pollen functionality and was more pronounced among wild grapevine individuals (male with fertile pollen vs. female with sterile pollen) due to differences in flower morphology and functional dioecy [8].

Only recently, palynological and archaeobotanical research has emphasized the importance of studying grapevine pollen dimorphism and pointed to a possible new investigation strategy into the history of wild and domesticated grapevine [8]. The advancement of electron microscopy (transmission and scanning) has provided a powerful technique for studying pollen morphology [9], prior to which it was not possible to reliably distinguish the pollen grains of domesticated and wild compartments [22]. Nevertheless, even with high-resolution techniques, in the case of *Vitis vinifera* spp., taxonomic identification would require more than just pollen evidence [8]. Studies on the fruit-set in grapevine have highlighted the importance of pollen morphology characterization, particularly pollen wall formation [14], the presence and structure of pollen pores and germination apertures [16], or specific morphological traits such as pollen type, exine ornamentation, polar axis (P), equatorial diameter (E) and P/E ratio [13].

Pollen grains are differently equipped to accomplish their role in pollination and fertilization, being aerodynamic, small-sized, light and shaped to fit their ‘target’ [8,23]. In rare instances, pollen shape and dimorphism may be observed from a perspective of unfunctionality or sterility. Male sterility in female individuals is associated with a lack of pollen apertures. Regions at the pollen surface with little or no exine deposition, and one of the most characteristic and well-defined structures of the pollen surface [24], are crucial in reproduction as a site for initial pollen tube growth [25]. Two predominant morphotypes are observed based on the apertures in the grapevine. Most of the studied grapevine cultivars and the male individuals of wild grapevine have 3-colporated pollen grains. Some female cultivars and wild female grapevine individuals have inaperturate pollen without colpi, which might be responsible for abnormal flower morphology producing less or irregular yields [8,20,25]. The pollen of female wild grapevine, despite viability, lacks germination and is considered sterile [25], being also smaller compared to the inaperturate pollen of cultivars [12,26]. Fertile and sterile pollen differ in microspore cell wall composition rather than in architecture and shape. In wild female individuals, microspores seem to have undergone normal microsporogenesis, similar to that in male individuals. Only upon microspore release from the anthers does the difference become obvious [4]. There are a few candidate genes associated with pollen suppression in grapevine [27], of which the gene involved in aperture formation, INP1 (inaperturate pollen 1), drew greater attention in genetic studies [7].

Recently, a project aiming to study the genetic and phenotypic diversity of wild grapevine in Croatia has been launched. Several natural populations of wild grapevine in Croatia have been identified using SSR markers [28], but their morphometric characteristics are still poorly studied. Only a few papers [4,8,12,29] have dealt with the pollen morphology of wild grapevine using scanning electron microscopy, despite the fact that flower type and pollen function constitute the basic taxonomic distinction between domesticated and wild grapevine.

Pollen formation in wild grapevine is of great interest from a biological, archaeological and breeding perspective. Therefore, the aim of this study was to reveal the microstructure of wild grapevine pollen grain in Croatia to widen the current knowledge on flower and pollen morphology.

## 2. Results

### 2.1. Pollen Microstructure

**Figure 1:**
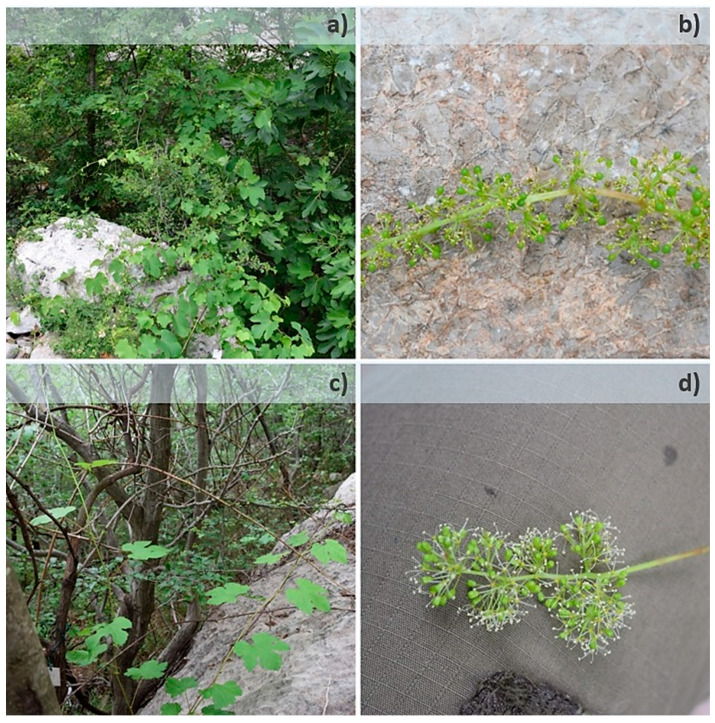
Two *V. sylvestris* individuals from the same population as an example of difference in flower morphology: female Pak10 (**a**,**b**) and male Pak21 (**c**,**d**). Each individual captured in natural habitat with closer view to its flower type.

**Figure 2:**
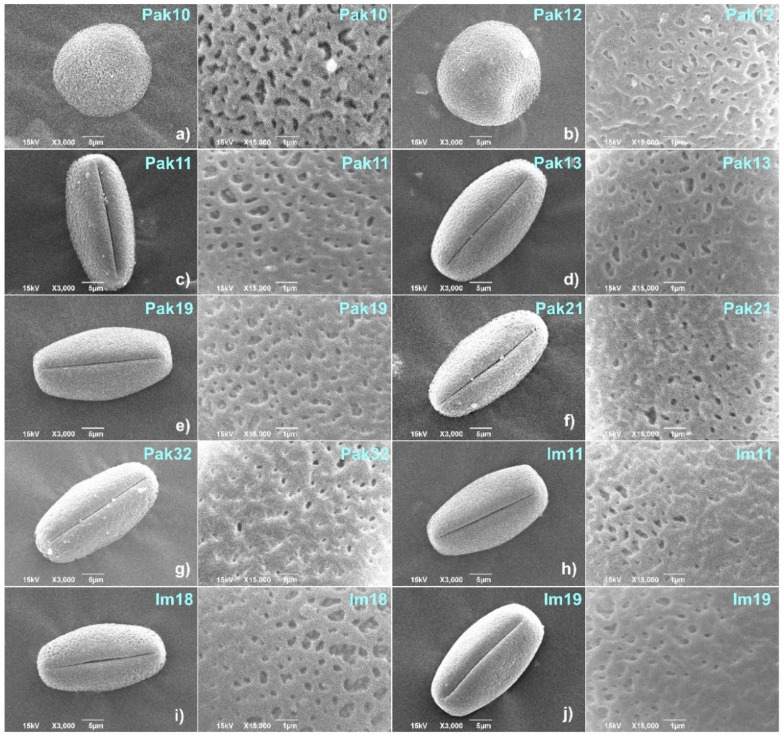
Equatorial view and detailed exine pollen surface in ten *V. sylvestris* genotypes (two female (**a**,**b**) and eight male (**c**–**j**). Each individual is represented by two images in line showing whole pollen grain (scale bar: 5 µm) and exine pattern (scale bar: 1 µm), respectively.

From the 10 different *V. sylvestris* genotypes analyzed in this study, eight had functionally male flowers, and two had functionally female flowers (Figure 1). Regardless of the flower type or sampling location, all pollen grains were isopolar and radially symmetrical, with foveolate-perforate exine ornamentation. Significant differences were observed in the dimensions and shape of the pollen from male and female individuals (Table 1, Figure 2).

The pollen of male *V. sylvestris* was 3-colporate and prolate (P/E ratio: 1.78–1.93), whereas that of the female was inaperturate: no colpi or apertures, spheroidal to subprolate in shape (P/E ratio: 1.12–1.15). 

In this study, the polar axis ranges from ~25.0 (female Pak12) to ~30.60 µm (male Im19). The equatorial axis ranges from ~15.2 µm (male Pak21) to 22.5 µm (female Pak10). The equatorial axis had the lowest significant variability among the studied individuals, but significantly differed in male and female individuals. The mean values for the P/E ratio ranged from 1.12 (female Pak10) to 1.93 (male Pak21).

The number of perforations per µm^2^ at the exine surface varied significantly, from 29.67 to 50.67 (both values for male individuals), and two female individuals also had a significant difference in this trait (Pak12 having a significantly higher number of perforations than Pak10). The lowest perforation length (330.14 nm) was observed for male Im11, and the highest for male Pak 11 (506.38 nm). Perforation width was the lowest for Im11 (206.54 nm) and the highest for Im18 (328.86 nm). The highest significant difference in colpi measurements for male individuals was observed between Pak11 and Im19, though colpus width did not vary significantly among the tested genotypes. The mesocolpium width was significantly higher in the case of Pak11 and Pak32 (~10.9 µm, each) compared to Im18 (9.9 µm).

### 2.2. Groupings of Individuals Based on Pollen Characteristics

**Figure 3:**
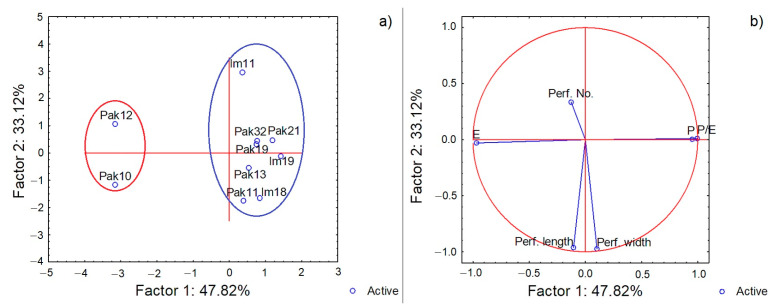
Two-step PCA representation of *V. sylvestris* genotypes: (**a**) Projection of points approximate the differences between samples. Nearby points represent samples with similar patterns, i.e., male individuals with 3-colporate pollen are grouped to the right in the plot (blue circle); female individuals with inaperturate pollen are grouped to the left in the plot (red circle). (**b**) Vectors approximate phenotypic trait. Highly correlated traits point in roughly the same direction. The longer the arrow, the stronger the correlation. The polar axis (P) and P/E ratio have strong positive correlation opposite to equatorial axis (E) length, with strong negative correlation toward P and P/E. Grouping of male individuals is mainly explained by P and P/E, and female individuals were negatively correlated with male individuals, being differentiated mainly by pollen width (E).

PCA analysis (colpi values excluded) (Figure 3a,b) reflected the morphological differentiation represented in Table 1. The first two axes explained 80.94% of the overall variation in the studied set and separated male *V. sylvestris* with 3-colporate pollen grains on the right side from female individuals with inaperturate pollen grains on the left side of the 2-D plot. Two female individuals (with inaperturate pollen) were distinguished mainly by the equatorial axis length (Figure 3a). The leading phenotypic traits for this sample set were pollen length (P) and grain size-based gradient P/E having a strong but negative correlation with pollen width (E), together explaining 47.82% of the total variation along the first principal component. Exine ornamentation traits (perforation width and length) were strongly correlated to each other along the second principal component, but were independent from pollen dimensions (P, E and P/E) due to a very weak correlation. The number of exine perforations, despite significant differences observed among the genotypes, showed the weakest correlation to other traits (shortest vector).

**Figure 4:**
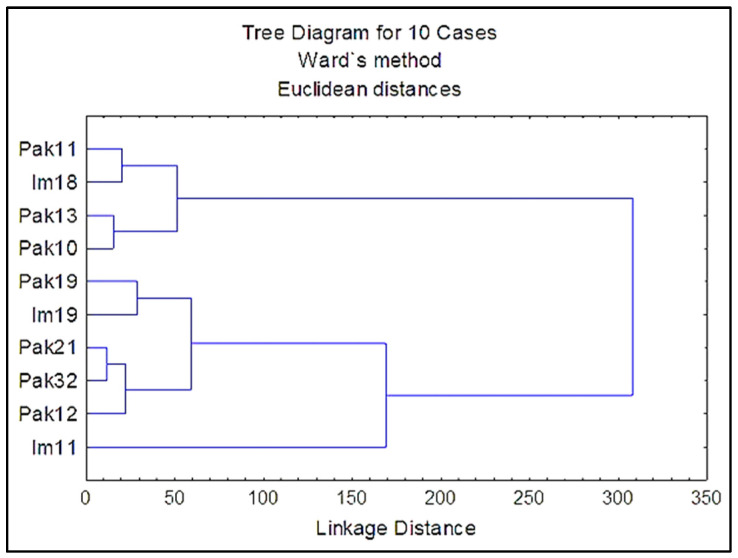
Ward’s dendrogram of *V. sylvestris* genotypes constructed based on the Euclidean distance matrix from six studied morphological characteristics of pollen grains: polar axis (P), equatorial axis (E), P/E ratio, number of exine perforations, perforation width and perforation length, in Statistica.

Ward’s clustering method grouped individuals into two main subclusters (Figure 4). This hierarchical clustering based on morphological traits (colpi values excluded) showed no particular grouping regarding the flower type or geographical origin of the individuals.

### 2.3. Flower Determination with APT3 Marker

**Figure 5:**
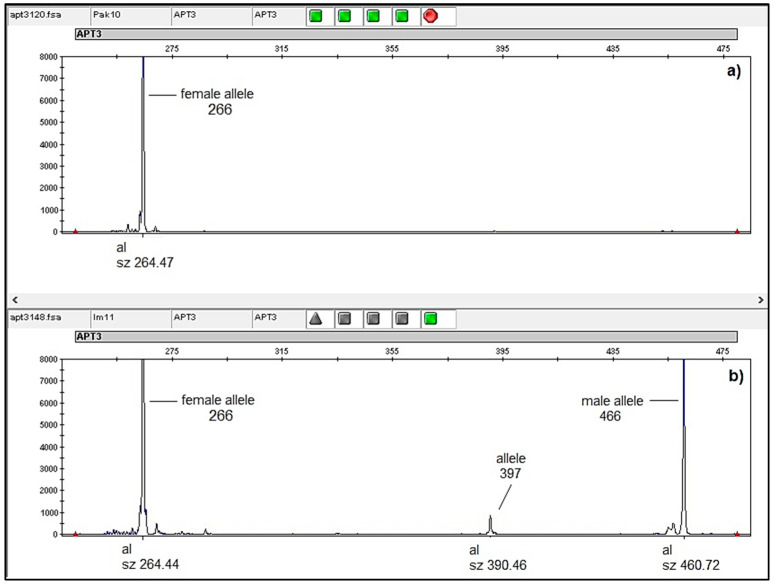
Screenshot of electropherogram (GeneMapper 6.0, Applied Biosystems, Waltham, MA, USA) displaying flower polymorphism at APT3 marker: (**a**) female individual Pak10, homozygous for allele 266 linked to female *V. sylvestris* [31], (**b**) male Im11 individual with the most frequent allelic pattern 266-397-466 at APT3.

Of eight analyzed male (‘M’) individuals, all had the allelic combination 266-397-466, except for one (Pak13) with a 266–466 combination at the APT3 marker. Both female (‘F’) individuals were homozygous at APT3, having a 266–266 allele (Figure 5, Table A1).

## 3. Discussion

### 3.1. Pollen Dimorphism

The microstructure of pollen from *V. sylvestris* in Croatia was revealed by scanning electron microscopy. Ten individuals from two populations were considered. The population in National Park Paklenica has been strictly protected for over 70 years; it belongs to the mountainous region with a shady forest habitat, with plenty of supporter plants for *V. sylvestris*. The population in Imotski is located further south, near the urban centers and with no strict protection of the site, fewer *V. sylvestris* individuals, poor supporter flora and soil characteristics, with dynamic water oscillations during the year [32]. Despite the population differences, pollen morphology and shape was not associated with population origin (Figure 3 and Figure 4), though for the 3-colporate pollen of *V. sylvestris* from Turkey, although similar in morphology, a slight difference was observed in the shape at various localities [29]. The results for Croatian *V. sylvestris* are in accordance with findings from the Danube River area that showed a similarity in pollen morphological characteristics observed under light microscopy—a low variation in pollen size (P × E) despite long distances between populations [26]. An obvious difference was recorded in pollen between male and female *V. sylvestris*. Previous results on *V. sylvestris* revealed greater diversity among *V. sylvestris* itself rather than between *V. sylvestris* and *V. vinifera* [12]. This sample set, although small-sized regarding the number of individuals, encompassed more male than female individuals, reflecting male–female representations in natural habitats previously observed in Croatia and elsewhere [33,34,35]. The male predominance might correspond to the abundant pollen production and fertility of male pollen in wild populations [36]. 

All the pollen grains in this study, regardless of flower type, were radially symmetrical and isopolar, which is consistent with the pollen morphology of *V. sylvestris* studied in Turkey [29]. The exine ornamentation for all grains in this study was foveolate-perforate. Inceoğlu et al. [29] reported on foveolate-rugulate and distinctly reticulate exine ornamentation at different pollen sites under SEM. Slight variations in ornamentation were observed at different sites of the same pollen grain or due to different sample preparation and the observation method used, as seen in studies [8,29]. The exine perforation observed under SEM in this study is in line with [29 cit. Faegri and Iversen 1964], as they reported that the exine sculpturing of *Vitis* was reticulate and foveolate-perforate under light microscopy. The entire exine sculpturing during pollen development might be affected by the expression of the INP1 gene [23]. 

The pollen grains of the male *V. sylvestris* in this study were tri-colporated and prolate in shape. This is usually observed in *Vitis* pollen, both in wild male and hermaphroditic cultivars [8,37]. Three-colporate pollen is linked to hermaphroditic flowers [20]. The prolate shape of pollen grains, with subtle variations, was observed in other *V. sylvestris* material [8,12,29]. Recently, prolate pollen was more frequently found in archaeological pollen than in modern pollen grains in Italy [8].

The pollen grains of the female *V. sylvestris* in this study were more oval (spheroidal to subprolate) than the pollen grains of the male individuals, and inaperturate. Quantitative traits such as pollen length (P), P/E ratio, number of perforations and colpus length were most informative for this sample set. Pollen length (P) varied significantly between the male–male and male–female individuals. Pollen width (E) did not vary significantly in this study, except for successfully differentiating males from females in accordance with their substantially different pollen shapes (Figure 2 and Figure 3).

The pollen grains in this study were slightly larger in dimension than the ones observed in Western European *V. sylvestris* and in Italy [8,12]. This might be due to differences in pollen measurement methods—[8] used a light microscope, whereas the SEM method was used in this study—or possibly due to differences in sample sizes [38]. The pollen grains of female individuals in a previous study were found to be slightly thicker than the pollen grains of male individuals [8]. The pollen in *Vitis* is heavier compared to some species that have a light pollen grain to enable wind pollination. Though *V. vinifera* mostly depends on wind pollination, in *V. sylvestris*, pollination by insects seems to be even more important [2]. The inaperturate pollen of female individuals, with an excess deposition of sporopollenin in their oblate and a closed structure lacking germination spores, is associated with being a reward for pollinators [8], and could possibly explain the low pollen dispersal efficiency in *Vitis*.

The APT3 marker used in this study to genetically verify flower dimorphism in *V. sylvestris* clearly separated female from male individuals and confirmed the flower type determined by visual evaluation. Female individuals had typical female structures and were homozygous at the APT3 marker (266 allele) (Figure 5) according to [31]. Male individuals all had typical male flower phenotypes and the allelic combination 266-397-466 at the APT3 marker, except for one individual (Pak13) that had a 266-466 allelic pattering. With the APT3 marker, it was possible to differentiate female individuals, but it was not possible to reliably distinguish males from hermaphrodites, which are occasionally found in natural habitats. Therefore, the APT3 marker was complementary to phenotypic observation for reliable identification of the flower type for *V. sylvestris*.

The multivariate analyses used in this study were both informative, especially PCA, which clearly differentiated male from female individuals based on morphological pollen data and revealed the phenotypic traits that contributed to pollen differentiation between male and female *V. sylvestris*.

### 3.2. Pollen Apertures

A female flower with a normally developed pistil and stamens, but short, curved or reflexed filaments, is related to the production of inaperturate pollen that seems to be sterile due to a lack of apertures preventing pollen germination [8]. Inaperturate pollen might also explain the poor fruit-set in *V. sylvestris* [8]. It is unknown whether the absence of pollen apertures in female individuals is sufficient to cause sterility in the grapevine [27]. Gallardo et al. [12] reported that inaperturate pollen in *V. sylvestris* is viable but lacking germination, and cultivars produce less fruit [12,20] or are sterile [39]. A recent study on the female cultivar ‘Malbo Gentile’ showed <5% of pollen with apertures, and inaperturate pollen, despite high viability (>90%), was unable to germinate. An in vitro germination test excluded the possibility that this cultivar produces cryptoaperturate pollen (pollen with an endoaperture that is not apparent in the surface view), indicating that pollen sterility is related to a physical barrier for pollen tube germination [8,40].

Female cultivars with inaperturate pollen are common, particularly among proles orientalis [41]. The cultivation of female cultivars is continually declining in modern vineyards due to fruit-set issues. Inaperturate pollen in cultivars is usually larger than in *V. sylvestris* individuals [12], similar to other results [8]. In a recent archaeological study, this type of pollen was proposed as a putative indicator of female *Vitis sylvestris* [8]. Interestingly, inaperturate pollen found in some species is correlated with an aquatic and moist, or nutrient-poor, habitat [40].

Other than the absence of apertures and pollen morphology itself, sterility in *V. sylvestris* might be related to poor nutrition uptake, e.g., zinc and boron at natural sites that are directly involved in the flower specification and fruit-set [21,39,42].

Alva et al. [39] reported the presence of various morphotypes of abnormal pollen in cultivars, all of which failed to germinate. The germination of abnormal pollen grains when supplemented with boron had only a minor effect compared to the treatment in cultivar with normal pollen, pointing to functional differences between normal and abnormal pollen. An excess of sporopollenin and exine deposition could result in infertile pollen [43]. A large deposition of these elements possibly leads to small and oval pollen grains lacking pores [12]. According to [30], two female individuals from the same population in this study differed significantly in the number of exine perforations and in pollen shape, which might be related to the inaperturate pollen specificities described.

The pollen grains of the male individuals in this study differed mostly in P/E ratio, number and length of perforations and colpus length. In *Arabidopsis*, a shorter aperture length proved to be related to lower transcript levels of the INP1 gene. Although INP1 is essential for aperture development, it is not the main factor defining aperture number, positions and morphology [24].

## 4. Materials and Methods

### 4.1. Sampling Locations and Plant Material

**Figure 6:**
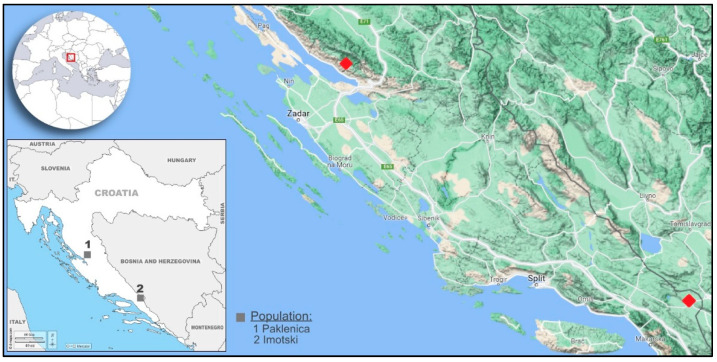
Two natural populations of *V. sylvestris* individuals analyzed in Croatia.

The studies were carried out with the pollen of *V. sylvestris* collected in 2018 from 10 individuals (eight male and two female), from two populations that were ~175 km apart (Table 2, Figure 6). The population of National Park Paklenica, located in the mountainous region of Velebit in northern Dalmatia, has been strictly protected since 1949, with no or minimum human impact. Paklenica belongs to humid *V. sylvestris* forest habitats in Croatia, with vertically growing *V. sylvestris* lianas in the shade along both sides of the Paklenica stream and rich supporter-plant flora. The southern population in Imotski, in central Dalmatia, by contrast, is in the vicinity of urban centers. *V. sylvestris* grows horizontally on the rocky shores of the lake, receiving more sunlight during vegetation, with poor supporter vegetation, shallow, erosion-prone soil and pronounced yearly lake water oscillations, from complete coverage of individuals in water to a completely dry habitat [32]. Sampling was done during full bloom (in June) by excising *V. sylvestris* inflorescence and placing it in a clean and dry 50 mL polypropylene tube with a desiccator containing silica gel for each genotype, and storage at −20 °C.

### 4.2. Scanning Electron Microscopy of Pollen

Samples were transferred to the laboratory of the Faculty of Agriculture (Belgrade-Zemun, Serbia) for further pollen analyses. The analyses in this study were conducted according to [44,45,46]. Anthers were isolated from the flowers, collected in small vials and stored at 3–5 °C in a silica gel desiccator until analysis. After dehydration, the pollen was mounted for analysis using a fine brush on aluminum stubs covered with double-sided transparent tape and coated with a 0.02 μm-thick gold layer in a sputter coater BAL-TEC SCD 005 (Capovani Brothers Inc., Scotia, NY, USA). Observation was carried out at 15 kV with a scanning electron microscope (SEM) JEOL JSM-6390LV (Tokyo, Japan) at a magnification of 3000× (whole grain) and 15,000× (exine pattern). At least 30 randomly collected pollen grains per genotype were analyzed and presented as measurements of three replicates per genotype (10 pollen grains per replicate). The traits characterized were pollen size and shape (length, width and length/width ratio), length and width of the colpus, width of the mesocolpium and exine characteristics (the number of perforations per 25 μm^2^ of the exine surface, perforation length and width), polarity, symmetry, aperture and exine ornamentation. Pollen shape characteristics were classified according to [30].

### 4.3. DNA Extraction and Marker Analysis

The selective DNA marker APT3 Indel [31] was employed to confirm the phenotype of the flower type and to differentiate female from male (or hermaphrodite) individuals. 

Total genomic DNA was extracted from the young leaves using the NucleoSpin Plant II kit (Macherey-Nagel, Düren, Germany), with a working DNA concentration of 1 ng/μL.

Reaction mixtures of 5 µL were prepared using KAPA Fast Multiplex PCR Kit (2x) (Kapa Biosystems, Wilmington, MA), 100 pmol of fluorescently labeled (FAM) reverse primer and ~1 ng template DNA. PCR amplification was set up to three minutes initial denaturation at 95 °C, 30 cycles of denaturation at 95 °C (15 s), annealing at 60 °C (30 s), extension at 72 °C (30 s) and final extension for seven minutes at 72 °C. PCR amplificates were analyzed using capillary electrophoresis on an ABI 3730xl Genetic Analyzer (Applied Biosystems, Waltham, MA, USA) with GeneScan-LIZ 500 standard. The peaks were identified with GeneMapper 4.0 software (Applied Biosystems, Waltham, MA, USA).

### 4.4. Statistical Analysis

The mean value for pollen grains was reported with standard deviations. Data were statistically analyzed using a one-way analysis of variance (ANOVA). The significance of the differences between the mean values was determined using Tukey’s test and considered significant at *p* ≤ 0.05. Genotypes were clustered into similarity groups using Principal Component Analysis (PCA) and Ward clustering methods based on tested characteristics, except for colpi values (colpus length, width and mesocolpium width) that are lacking in female individuals. All statistical analyses were performed using the software package STATISTICA v. 14.0 (TIBCO Software Inc, Palo Alto, CA, USA).

## 5. Conclusions

Scanning electron microscopy revealed the structure of the pollen grains of male and female *V. sylvestris* in Croatia for the first time. The analyses clearly differentiated male from female pollen morphotypes, but no separation by population was observed. All pollen grains of *V. sylvestris* were isopolar, radially symmetrical and foveolate-perforate ornamented, which is in line with previous, similar studies. Two pollen morphotypes were observed: 3-colporate pollen with a prolate shape recorded in male individuals and inaperturate pollen with a spheroidal to subprolate shape in two female individuals that significantly differed in the number of exine perforations. Quantitative pollen dimensions described by polar axis, P/E ratio, number of perforations and colpus length were most polymorphic for the analyzed *V. sylvestris* set. PCA and cluster analysis was informative with regard to pollen sex dimorphism. This study opens the door for further studies on *Vitis* flower morphology and sterility issues by testing pollen germination and comparing the INP1 gene between hermaphroditic and female genotypes of *V. sylvestris*.

## Figures and Tables

**Table 1:** Morphometric data on pollen grains taken from 10 *V. sylvestris* genotypes (two female and eight male) from two different natural populations. Each value represents an average measurement ± standard deviation, based on a minimum of 30 pollen grains per genotype obtained by the scanning electron microscopy (SEM).

Genotype	Pollen Dimensions and Shape				Exine Perforations			Colpi Dimensions (Male Individuals)		
	P (µm)	E (µm)	P/E	Shape	No. of Perforations /25 µm^2^	Perforation Length (nm)	Perforation Width (nm)	Colpus Length (µm)	Colpus Width (µm)	Mesocolpium Width (µm)
Pak10	25.28 ± 1.1 ^c^	22.53 ± 1.13 ^b^	1.12 ± 0.01 ^d^	spheroidal	32 ± 1 ^ab^	486.91 ± 25.17 ^ab^	295.61 ± 22.85 ^a^			
Pak12	25 ± 2.14 ^c^	21.67 ± 1.26 ^b^	1.15 ± 0.05 ^d^	subprolate	47.67 ± 4.51 ^de^	412.98 ± 16.52 ^abc^	258.32 ± 9.9 ^ab^			
Pak11	27.84 ± 0.33 ^a^	15.59 ± 0.41 ^a^	1.78 ± 0.07 ^a^	prolate	33 ± 6 ^ab^	506.38 ± 70.14 ^b^	317.61 ± 62.54 ^a^	23.22 ± 0.67 ^a^	0.8 ± 0.05 ^a^	10.9 ± 0.24 ^a^
Pak13	28.23 ± 0.18 ^a^	15.35 ± 0.03 ^a^	1.84 ± 0.01 ^abc^	prolate	44.67 ± 0.58 ^cde^	483.68 ± 44.98 ^ab^	291.66 ± 12.81 ^a^	24.26 ± 0.18 ^abc^	0.72 ± 0.03 ^a^	10.23 ± 0.25 ^ab^
Pak19	29.41 ± 0.42 ^ab^	15.97 ± 0.25 ^a^	1.84 ± 0.05 ^abc^	prolate	50.67 ± 2.52 ^e^	445.03 ± 21.02 ^ab^	283.52 ± 15.83 ^ab^	25.07 ± 0.41 ^cd^	0.73 ± 0.16 ^a^	10.55 ± 0.12 ^ab^
Pak21	29.22 ± 0.11 ^ab^	15.17 ± 0.34 ^a^	1.93 ± 0.03 ^c^	prolate	35.33 ± 2.52 ^ab^	416.84 ± 21.29 ^abc^	268.15 ± 17.21 ^ab^	25.31 ± 0.16 ^cd^	0.79 ± 0.03 ^a^	10.3 ± 0.17 ^ab^
Pak32	28.9 ± 0.2 ^ab^	15.95 ± 0.33 ^a^	1.81 ± 0.05 ^abc^	prolate	29.67 ± 3.06 ^a^	406.48 ± 28.73 ^ac^	265.92 ± 31.29 ^ab^	24.36 ± 0.34 ^bc^	0.8 ± 0.05 ^a^	10.91 ± 0.41 ^a^
Im11	27.88 ± 0.22 ^a^	15.57 ± 0.27 ^a^	1.79 ± 0.04 ^ab^	prolate	39.67 ± 3.06 ^bcd^	330.14 ± 19.34 ^c^	206.54 ± 8.99 ^b^	23.81 ± 0.09 ^ab^	0.67 ± 0.02 ^a^	10.46 ± 0.43 ^ab^
Im18	28.49 ± 0.38 ^ab^	15.26 ± 0.26 ^a^	1.87 ± 0.05 ^abc^	prolate	36 ± 1 ^abc^	489.62 ± 24.18 ^ab^	328.86 ± 27.18 ^a^	23.73 ± 0.51 ^ab^	0.87 ± 0.08 ^a^	9.91 ± 0.09 ^b^
Im19	30.59 ± 0.23 ^b^	16.06 ± 0.12 ^a^	1.9 ± 0.01 ^bc^	prolate	36.33 ± 3.51 ^abc^	422.88 ± 5.56 ^abc^	295.09 ± 16.1 ^a^	25.92 ± 0.26 ^d^	0.71 ± 0.09 ^a^	10.59 ± 0.4 ^ab^

P: Polar axis (pollen length); E: Equatorial axis (pollen width). P/E: ratio of polar axis to equatorial diameter, according to [30]. Shape classification according to [30]. Mean values followed by different lowercase letters in columns represent significant differences according to Tukey’s test at *p* ≤ 0.05.

**Table 2:** *V. sylvestris* individuals analyzed in this study with information on their population and flower phenotype.

Population	Genotype	GPS Coordinates	Flower Phenotype
Paklenica	Pak10	44°18′ 028″ N 15°28′ 209″ E	F
Paklenica	Pak12	44°18′ 260″ N 15°28′ 318″ E	F
Paklenica	Pak11	44°18′ 042″ N 15°28′ 218″ E	M
Paklenica	Pak13	44°18′ 348″ N 15°28′ 452″ E	M
Paklenica	Pak19	44°18′ 351″ N 15°28′ 635″ E	M
Paklenica	Pak21	44°18′ 465″ N 15°28′ 705″ E	M
Paklenica	Pak32	44°17′ 919″ N 15°27′ 868″ E	M
Imotski	Im11	43°26′ 969″ N 17°12′ 524″ E	M
Imotski	Im18	43°26′ 994″ N 17°12′ 514″ E	M
Imotski	Im19	43°26′ 997″ N 17°12′ 526″ E	M

F, female; M, male.

## Data Availability

All data analyzed in this study are included in the article.

## Appendix A

**Table A1:** List of *V. sylvestris* individuals analyzed in this study with regard to their flower morphology and genetics. Flower phenotype was visually determined at natural habitats, and genetic background was revealed by selective DNA marker APT3 at intron 2 on chromosome 2.

Population	Genotype	Flower Phenotype	APT3		
Paklenica	Pak10	F	266	266	
Paklenica	Pak12	F	266	266	
Paklenica	Pak11	M	266	397	466
Paklenica	Pak13	M	266	466	
Paklenica	Pak19	M	266	397	466
Paklenica	Pak21	M	266	397	466
Paklenica	Pak32	M	266	397	466
Imotski	Im11	M	266	397	466
Imotski	Im18	M	266	397	466
Imotski	Im19	M	266	397	466

F, female; M, male.
